# An active learning-enabled annotation system for clinical named entity recognition

**DOI:** 10.1186/s12911-017-0466-9

**Published:** 2017-07-05

**Authors:** Yukun Chen, Thomas A. Lask, Qiaozhu Mei, Qingxia Chen, Sungrim Moon, Jingqi Wang, Ky Nguyen, Tolulola Dawodu, Trevor Cohen, Joshua C. Denny, Hua Xu

**Affiliations:** 1 Pieces Technologies Inc, Dallas, TX USA; 2 Department of Biomedical Informatics, Vanderbilt University, Nashville, TN USA; 3 Biostatistics, Vanderbilt University, Nashville, TN USA; 4 Medicine, Vanderbilt University, Nashville, TN USA; 5 School of Information, University of Michigan, Ann Arbor, MI USA; 6 Department of Health Sciences Research, Mayo Clinic, Rochester, MN USA; 7 School of Biomedical Informatics, The University of Texas Health Science Center at Houston, Houston, TX USA; 8 Nursing, The University of Texas Health Science Center at Houston, Houston, TX USA

## Abstract

**Background:**

Active learning (AL) has shown the promising potential to minimize the annotation cost while maximizing the performance in building statistical natural language processing (NLP) models. However, very few studies have investigated AL in a real-life setting in medical domain.

**Methods:**

In this study, we developed the first AL-enabled annotation system for clinical named entity recognition (NER) with a novel AL algorithm. Besides the simulation study to evaluate the novel AL algorithm, we further conducted user studies with two nurses using this system to assess the performance of AL in real world annotation processes for building clinical NER models.

**Results:**

The simulation results show that the novel AL algorithm outperformed traditional AL algorithm and random sampling. However, the user study tells a different story that AL methods did not always perform better than random sampling for different users.

**Conclusions:**

We found that the increased information content of actively selected sentences is strongly offset by the increased time required to annotate them. Moreover, the annotation time was not considered in the querying algorithms. Our future work includes developing better AL algorithms with the estimation of annotation time and evaluating the system with larger number of users.

## Background

Named entity recognition (NER) is one of the most fundamental tasks for many clinical natural language processing (NLP) applications. Although machine learning (ML) based NLP systems could achieve high performance, they often require large numbers of labeled data, which is expensive to obtain with the use of domain experts in annotation. To minimize the cost while optimizing the performance, many studies in general English NLP have shown that pool-based AL framework [1] could be a cost-effective solution to build the high-quality ML based NLP models with smart sampling strategies. The NLP tasks enhanced by AL include word sense disambiguation (WSD) [2], text classification [3], and information extraction [4]. Recently, several studies have also presented the effectiveness of AL to NLP tasks in the clinical domain. Figueroa et al. [5] validated AL algorithms in five medical text classification tasks to reduce the size of training sets without losing the expected performance. Chen et al. applied AL on multiple biomedical NLP tasks, such as assertion classification for clinical concepts [6], supervised WSD in MEDLINE [7], high-throughput phenotype identification tasks using EHR data [8], and clinical NER [9]. All the above draws a conclusion that AL, compared to passive learning based on random sampling, could induce annotation cost reduction while optimizing the quality of the classification model.

Most of these AL studies were conducted in a simulated setting, which assumes that annotation cost for each sample is identical. In reality, however, annotation cost (i.e. the time required to annotate one sample by an annotator) can be very different from one sample to another, or from one annotator to another. The estimated cost savings by AL in simulated studies may not be applicable in reality. Settles et al. [10] conducted a detailed empirical study to assess the benefit of AL in terms of real-world annotation costs and their analysis concludes that a reduction in the number of annotated sentences required does not guarantee a real reduction in cost. Therefore, to better understand how AL works within the real time annotation process and to demonstrate the utility of AL in real-world tasks in the clinical domain, we should integrate AL technologies with annotation systems and validate its effectiveness by recruiting users to conduct real-world annotation tasks.

In the user study, we built an active learning enabled annotation system for clinical NER. Using this system, we compared the performance of AL against random sampling in the user study. Our results show that AL did not guarantee less annotation time than random sampling across different users, at a given performance point of the model. We then discuss other findings in our experiments and the limitations of the proposed *CAUSE* (*Clustering And Uncertainty Sampling Engine*) method, with suggestions to future improvements.

## Methods

The clinical NER task in this study was to extract problem, treatment, and lab test concepts from clinical notes. We first developed an AL-enabled annotation system, which iteratively builds the NER model based on already annotated sentences and selects the next sentence for annotation. Multiple new querying algorithms were developed and evaluated using the simulated studies. For the user study, the best querying algorithm from the simulation was implemented in the system. Two nurses were then recruited and participated in the real-time annotation experiments using the system for both *CAUSE* and *Random* modes.

### Development of the active learning-enabled annotation system

Practical AL systems such as DUALIST [11] have been developed to allow user and computer to iteratively interact for building supervised ML models for different NLP tasks, such as text classification and word sense disambiguation. For sequence labeling tasks such as NER, however, there is no existing interactive system available. In this study, we designed and built a system named *Active LEARNER* (also called *A-LEARNER*), which stands for *Active Leaning Enabled AnnotatoR for Named Entity Recognition*. To the best of our knowledge, it is the first AL enabled annotation system serving clinical NER tasks.

The front end of *Active LEARNER* is a graphic user inference that allows users to mark clinical entities in a sentence supplied by the system using a particular querying engine. In the back end, the system iteratively trains CRF models based on users’ annotations and selects the most useful sentences based on the querying engine. The system implements a multi-thread processing scheme to allow a no-waiting annotation experience for users.

*Active LEARNER* uses the unlabeled corpus as the input and generates NER models on the fly, while iteratively interacting with the user who annotates sentences queried from the corpus. The *Active LEARNER* system consists of three components: 1) the annotation interface, 2) the ML-based NER module, and 3) the AL component for querying samples. For the annotation interface, we adopted the existing *BRAT* system, a rapid annotation tool developed by Stenetorp P et al. [12]. We modified the original *BRAT* interface to allow users to mark entities more efficiently. The ML-based NER module was based on the *CRF* algorithm implemented by *CRF++* https://taku910.github.io/crfpp/, as described in [13]. The AL component implemented some existing and novel querying algorithms (described in later sections) using a multi-thread framework. More details of the *Active LEARNER* system are described below.

#### System design

**Fig. 1:**
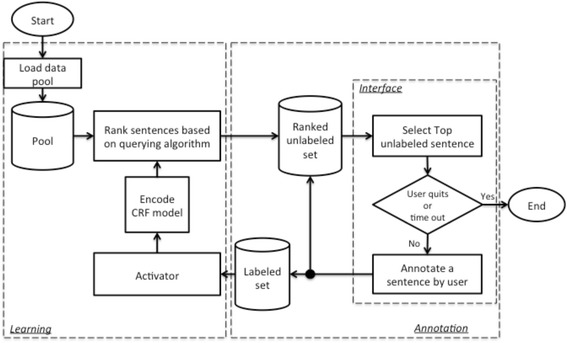
Workflow of Active LEARNER

Figure 1 shows the workflow of the *Active LEARNER* system. Once the system starts, the pool of unlabeled data is loaded into the memory. At the initial iteration or before the CRF model is generated, all sentences are randomly ranked. The top sentence in the ranked unlabeled set is queried and displayed on the interface. The user then can highlight clinical entities in the sentence via the labeling function on the interface (*annotation process*). When the user submits the annotated sentence, the *labeled set* and the *unlabeled set* are updated and the *learning process* is activated based on activation criteria. When the *learning process* is complete, the ranked unlabeled set is updated while the next sentence is available for annotation.

What annotator could do after one annotation is submitted and before the *learning process* is complete? To avoid such delay in the workflow, we parallelize the *annotation process* and *learning process*. In the *annotation process*, the black circle in Fig. 1 splits the flow into two that run simultaneously. One sub-flow runs back to the *ranked unlabeled set* and interface. Therefore, the user can immediately read the next sentence on the interface right after the annotation for one sentence is submitted. The other sub-flow adds the newly annotated sentence to the labeled set, which is then pushed to the *learning process*. A new *learning process* will be activated if the encoding or querying process is not busy and the number of newly annotated sentences is greater or equal to a threshold (five in our study), which is for the update frequency control. When the *learning process* is activated, it runs in parallel with the *annotation process* and it updates the *ranked unlabeled set* whenever the new rankings are generated. This design allows a user to continuously annotate the top unlabeled sentence from the ranked list, which is generated in either the current or previous *learning process*. The program is stopped when the user either clicks the quit button or a pre-set cutoff time runs out.

Specifically, the *learning process* includes CRF model encoding based on the current labeled set and sentence ranking by the querying engine. The CRF model encoding is straightforward; however, it could take time to rebuild the CRF model when the labeled data set gets bigger. Sentence ranking consists of two steps: 1) CRF model decoding, which is to make predictions for each unlabeled sentence based on the current model; and 2) ranking sentences by the querying algorithm, which considers both the probabilistic prediction of each sentence from the first step, and other information about the unlabeled sentences (i.e. sentence clusters).

### Querying methods

In our previous study [9], we have described multiple AL querying algorithms and shown that uncertainty based sampling methods are more promising than other methods to reduce the annotation cost (in terms of the number of sentences or words) in the simulated studies. In this study, we further developed a novel AL algorithm that considers not only the uncertainty but also the representativeness of sentences. The AL methods were compared to a passive learning method based on random sampling (*Random*) in both the simulation and the user studies.

Uncertainty based sampling methods are promising for selecting the most informative sentences from the pool for the clinical NER modeling. However, these methods could not distinguish the most representative sentences with respect to their similarity. As similar sentences could share very close uncertainty scores, the batch of the top ranked sentences could possibly contain multiple similar sentences with repeated clinical concepts. Before the *learning process* is completed, these concepts may be annotated more than once in these similar sentences during the *annotation process*. Obviously, annotating such similar sentences is not the most efficient for building NER models although these sentences are most informative.

Here, we propose the *Clustering And Uncertainty Sampling Engine* (*CAUSE*) that combines clustering technique and uncertainty sampling to query both informative and representative sentences. This method guarantees that the top ranked sentences in a batch are from different clusters and thus dissimilar with each other.

The algorithm of *CAUSE* is described as the following:

#### Input

(1) Clustering results of sentences; (2) Uncertainty scores of sentences; (3) Batch size (x);

#### Steps

(1) Cluster ranking: score each cluster based on the uncertainty scores of sentences and select the top x cluster(s) based on the cluster scores, where x is the batch size; (e.g. the score of a cluster could be the average uncertainty score of sentences in this cluster.)

(2) Representative sampling: in each selected cluster, find a sentence with the highest uncertainty score as the cluster representative.

#### Output

x cluster representative sentences in the order of their cluster ranking.

#### Initial sampling

When the NER model and uncertainty scores of sentences are not available, we used random sampling to select a cluster and the representative within the selected cluster.

The following sections describe how exactly the *CAUSE* algorithm was implemented in this study.

#### Sentence clustering with topic modeling

Clustering is a required pre-processing step in *CAUSE* for the pool of data to be queried. The clustering process consists of Latent Dirichlet Allocation (*LDA*) [14], a topic modeling technique, for feature generation, and affinity propagation (*AP*) [15] for clustering. In this clinical concept extraction task, we need to group semantically similar sentences together. We applied a C implementation of *LDA* (*LDA-C*) [16] to extract the hidden semantic topics in the corpus of clinical notes. Since using document-level samples for topic modeling could generate more meaningful topics than sentences, we ran *LDA* topic estimation on the entire dataset from the 2010 i2b2/VA NLP challenge (826 clinical documents). Given the K estimated topics, the *LDA* inference process was performed to assign probabilistic values of topics for every sentence. Eventually, each sentence was coded in a K dimensional vector with a probability at each of the K topics as value. Cosine similarity was used to calculate the similarity between every sentence pair. Next, we applied a python package of *AP* http://scikit-learn.org/stable/modules/generated/sklearn.cluster.AffinityPropagation.html that takes the M x M pair-wise similarity matrix as the input and outputs the clustering result for the M sentences.

#### Cluster ranking

Each cluster is assigned a score based on one of the following schemas: (a) Maximum uncertainty cluster sampling (MUCS): assign the cluster the highest uncertainty score among all the sentences in the cluster; (b) Average uncertainty cluster sampling (AUCS): assign the cluster the average uncertainty score from all the sentences in the cluster; (c) Random cluster sampling (RCS): assign the cluster a random score (assuming that each cluster is equally important). According to our experiments, AUCS performed the best in terms of learning curve performance. The cluster with a higher score will be ranked higher among all clusters, thought to contribute most to the NER modeling.

#### Representative sampling

From the top ranked cluster, we select the sentence that has the highest uncertainty score as the representative of the cluster. We also find the representative sentences from the second ranked cluster, third ranked cluster, and so on. We keep sampling until the batch is filled up with representatives. The ranking of the representatives follows the ranking of their clusters. We assume that the number of clusters is greater than or equal to the batch size so that the batch cannot contain more than one sentence from a cluster.

The assumption here is that cluster representative sentences can improve the NER model by helping identify entities from other sentences in the same cluster. Table 1 shows an example of a cluster that contains multiple sentences about medications. The cluster representative is the first sentence, where “Dulcolax” is tagged as the medication treatment. When the NER model is trained on this annotated cluster representative, the model could identify other medications (e.g. “Amaryl”, “Nortriptyline”, “Metformin”, etc.) from additional sentences in the same cluster based on their similar context (e.g. “mg”, “p.o.”, and “q.”) as the cluster representative.

**Table 1:** An example of a cluster that contains multiple sentences about prescription

Cluster representative	Sentences in a cluster
X	14. Dulcolax 10 mg p.o. or p.r. q. day p.r.n.
	9. Amaryl 4 mg p.o. q. day .
	3. Nortriptyline 25 mg p.o. q. h.s.
	2) Metformin 500 mg p.o. q. 8 h .
	…

### The user study

The user study is to evaluate the performance of AL versus passive learning in the real-world annotation processes for building NER models. The annotation cost in the user study is the actual annotation time by an annotator; the annotations (i.e. clinical entities) are done by users on-the-fly, instead of from a pre-annotated gold standard. Two nurses are recruited to use *Active LEARNER* to annotate sentences to evaluate both *CAUSE* and *Random* modes.

We understand that there are many human factors influencing the user study, such as annotation speed and annotation quality, in addition to querying methods. To make the results of two methods comparable, we rigorously trained two users in the annotation process, to ensure they will perform consistently in both experiments. The user-training phase included the following steps:

#### Guided training

The first step of training is to study the annotation guidelines, which were generated by the 2010 i2b2/VA NLP challenge organizer https://www.i2b2.org/NLP/Relations/assets/Concept%20Annotation%20Guideline.pdf. Both nurses had some experience on similar chart review tasks. At the very first training session, the NLP expert discussed the annotation guidelines with two nurses for 15–30 min, particularly focusing on the annotation boundaries of the clinical concepts. The next step was to review annotations sentence-by-sentence. The objective of this training session was to train users to be more familiar with both the annotation guidelines of the task and the *Active LEARNER* interface. Users were shown two interfaces on the left and right half of the screen. A user annotates a sentence on the left-side interface. When the annotation is finished, the user could review the i2b2 gold standard of the annotation for the same sentence on the right-side interface. If there was discrepancy between the user’s annotation and the gold standard, we discussed the possible reasons that support either gold standard or user annotation. A user could either stick to the original decision or change the annotation based on the discussion.

#### Practice

The practice process consists of two parts: 1) a shorter session with two to three 15-min of annotation; and 2) a longer session with four half-hour annotation, which was the same as the main user study discussed in the later section. The users conducted this part of training independently without breaks. We collected user’s annotation speed and annotation quality at each session so that we could track if the user achieved consistent annotation performance.

#### Warm up training section

In the second and third week of the user study, we conducted a shorter version of the training called warm up training. This served to refresh users on both annotation guidelines and interface usage. The warm up training also consisted of two parts. The first part was sentence-by-sentence annotation review. It took at least 15 min and up to 45 min. This part could be stopped when user was making annotations consistent with the i2b2 gold standard. The second part was two 15-min sessions of annotation. We used this opportunity to measure the user’s current speed and quality of annotation.

Table 2 shows the actual schedule we used in user study. Both users tested *Random* in week 1 and then *CAUSE* method in week 2. The reason to separate the user studies for two methods by a one-week gap is to allow users to forget the previous annotation. In each week, a user was required to go through a warm up training first, and then to complete the annotations of four half-hour sessions. The annotation time for each session was set to 30 min. A break of at least 10 min and up to 15 min was required between two sessions. During one session, each user was asked to continuously work without break in an isolated conference room with minimum interruption.

**Table 2:** Schedule of the user study

Time	Event	Task	Duration
Week 0	Guided Training	1. Annotation guideline review	30 min
		2. Sentence-by-sentence annotation and review using the interface	45 min
	Practice	1. Three quarter-hour sessions of annotation practice	45 min
		2. Four half-hour sections of annotation using *Random*, with 15-min break between sessions	3 h
Week 1	Annotation warm up training	1. Sentence-by-sentence annotation and review using the interface	15 - 30 min
		2. Two 15 min sessions of annotation practice	30 min
	Main study for method Random	Four 30 min sessions of annotation using Method 2	3 h
		15-min break between sessions	
Week 2	Annotation warm up training	1. Sentence-by-sentence annotation and review using the interface	15 - 30 min
		2. Two 15 min sessions of annotation practice	30 min
	Main study for method CAUSE	Four 30 min sessions of annotation using Method 2	3 h
		15-min break between sessions	

### Datasets

We used the annotated training corpus from the 2010 i2b2/VA NLP challenge [17]. The clinical named entity recognition task is to identify the medical concepts of problem, treatment, and lab test from the corpus. The dataset with 20,423 unique sentences was randomly split into five folds, each of which has either 4,084 or 4,085 unique sentences. In the simulation, we performed 5-fold cross validation so that four out of five folds were used as the pool of data to be queried and the remaining fold was the independent test set for evaluation. In the user study, we used fold 1 with 4,085 unique sentences as the independent test set and the remaining 16,338 unique sentences as the pool for data querying. In the annotation warm up training, the reviewed sentences are from the independent test set. Table 3 shows the characteristics (counts of sentences, words, and entities, words per sentence, entities per sentence, and entity density) in five folds of the dataset and the pool of querying data.

**Table 3:** Characteristics (counts of sentences, words, and entities, words per sentence, entities per sentence, and entity density) in five folds of the dataset and the pool of querying data

	Sentence count	Word count	Entity Count	Words per sentence	Entities per sentence	Entity density^a^
Fold 1	4,085	44,403	5,395	10.87	1.32	0.25
Fold 2	4,085	45,588	5,183	11.16	1.27	0.24
Fold 3	4,084	45,355	5,201	11.11	1.27	0.24
Fold 4	4,085	45,141	5,263	11.05	1.29	0.25
Fold 5	4,084	44,834	5,177	10.98	1.27	0.24
Pool (Fold 2 + 3 + 4 + 5)	16,338	180,918	20,824	11.07	1.27	0.24

^a^Entity density is the number of words of the entities divided by the total number of words

### Evaluation

In the simulation study, we used number of words in the annotated sentences as the estimated annotation cost. The learning curves that plot F-measures vs. number of words in the training set were generated to visualize the performance of different methods. For each method, the five learning curves from the 5-fold cross validation were averaged to general a final learning curve.

In the user study, actual annotation time was used as the annotation cost. We also generated the learning curves that plot F-measure vs. actual annotation time to compare both AL and passive learning. Moreover, there are many human factors that would affect the learning curve as well, such as user *annotation speed* and *annotation quality*. The most intuitive annotation evaluation metric to determine the *annotation speed* is the entity tagging speed (e.g. number of entities or entity annotations per minute). Obviously, if a user can contribute significantly more annotations in a given time, the learning curve of NER models could more likely be better regardless of querying methods. In addition, the *annotation quality*, which is measured by F-measure based on gold standard, is another important factor for training a clinical NER model. If we fix the *annotation speed*, higher *annotation quality* would more likely help build better NER models.

To globally assess different learning curves, we computed the area under the learning curve (ALC) as a global score for each method, which is calculated as the area under the given learning curve (*actual area*) divided by a *maximum area* that represents the maximum performance. The *maximum area* is equal to the ultimate cost spent in training (e.g. number of words in the final training set or the actual annotation time) times the best possible F-measure. Ideally, the best F-measure is 1.0. However, the NER models could never achieve perfect under only 120-min annotation. At this study, we used an F-measure of 0.75 as the best possible F-measure in 120-min annotation.

## Results

In the simulation, we evaluated methods of *Random*, *Uncertainty*, and *CAUSE* assuming same cost per word. Both *Uncertainty* and *CAUSE* utilized *LC* as the uncertainty measurement. The training process of *Uncertainty* and *CAUSE* started from 5 initially selected sentences based on random sampling. *CAUSE* used random cluster and representative sampling to select the initial 5 sentences. The batch size is 5 so that the model was updated with every additional 5 newly queried sentences. The AL process stopped at the point where there are as close as 7,200 words in the training set. This stopping criterion is to mimic the 120-min (7,200 s) long user study per method, assuming the user would annotate approximately one word per second from the user study results.

**Fig. 2:**
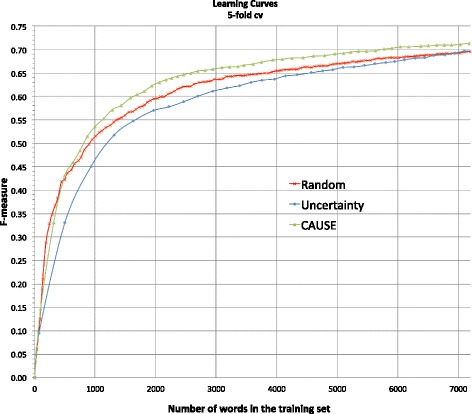
Simulated learning curves by 5-fold cross validation that plot F-measure vs. number of words in the training set for random sampling (*Random*), least confidence (*Uncertainty*), and *CAUSE* that used least confidence to measure uncertainty

Figure 2 shows the learning curves of *Random*, *Uncertainty*, and *CAUSE* in the same graph. Obviously, *CAUSE* outperformed *Random* and *Uncertainty* most of the time at all stages during the AL process. In terms of ALC score, *CAUSE* achieved 0.839, *Uncertainty* did 0.782, and *Random* did 0.812. At the point where there are ~7,200 words in the training set, *CAUSE* generated NER models with 0.713 in F-measure on average, while *Random* and *Uncertainty* achieved 0.696 and 0.697 in F-measure, respectively.

### User study results

For the user study, there are 16,338 unique sentences in the pool for querying and 4,085 unique sentences in the test set for evaluating NER models. Based on the simulated results, *CAUSE* performed better than *Uncertainty*. Therefore, we used *CAUSE* to represent AL in the user study and compared it with *Random* in the user study. The initial sentence selection schemas used in the user study were the same as the simulation. The batch size was set at 5, meaning the new *learning process* would be activated when there were at least 5 newly labeled sentences added to the *labeled set*.

Table 4 reports the assessment of annotation information from the main studies. Two users performed similarly with respect to *annotation speed* and *annotation quality* in the user studies of two methods (*Random* and *CAUSE*). It indicates that both users’ performances are stable and two methods could be comparable.

**Table 4:** User annotation counts, speed, and quality comparison in the 120-min main study

Users	Methods	Annotated entity count	Annotation speed (Entities per min)	Annotation quality (F-measure)
User 1	Random	945	7.88	0.82
	CAUSE	926	7.72	0.83
User 2	Random	882	7.35	0.81
	CAUSE	948	7.90	0.82

**Fig. 3:**
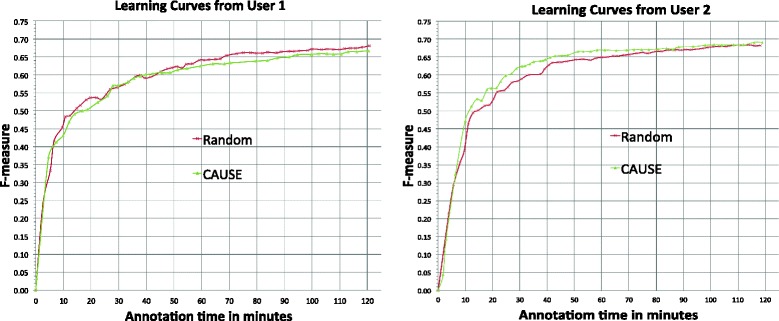
Learning curves of F-measure vs. annotation time in minutes by *Random* and *CAUSE* from user 1 and 2

Figure 3 show the learning curves of F-measure versus annotation time in minutes by *Random* (in week 1) and *CAUSE* (in week 2) from two users. The experimental results for the two users were different. *Random* performed better than *CAUSE* for user 1, while *CAUSE* was superior to *Random* for user 2.

Table 5 shows the ALC scores and F-measure of the final NER model at the end of 120 min annotation for *Random* and *CAUSE* from both users.

**Table 5:** Comparison between *Random* and *CAUSE* in ALC score and F-measure of the last model in the 120-min main study

User Index	Evaluated method	ALC scores	F-measure of models at 120 min
User 1	Random	0.812	0.680
	CAUSE	0.783	0.666
User 2	Random	0.820	0.682
	CAUSE	0.831	0.691

Table 6 and 7 summarize the characteristics of *Random* and *CAUSE* in each 120-min main study from both users. Both users annotated more sentences in the *Random* mode than that in the *CAUSE* mode, very likely due to shorter length of sentences selected by *Random*. Moreover, users seemed to read the words queried by *Random* faster than *CAUSE*. The entity number per sentence by *CAUSE* is about 3 times higher than that in *Random*. Entity density by *CAUSE* is also higher than that by *Random*.

**Table 6:** Characteristics of *Random* and *CAUSE* in each 120-min main study from user 1 and 2 (part 1)

User	Method	Annotated Sentences	Words in annotated sentences	Entities in annotated sentences	Words in entities
User 1	Random	655	8,023	945	1,915
	CAUSE	232	6,333	926	2,145
User 2	Random	651	7,325	882	1,952
	CAUSE	240	6,455	948	2,404

**Table 7:** Characteristics of *Random* and *CAUSE* in each 120-min main study from user 1 and 2 (part 2)

User	Method	Sentences per min	Words per sentence	Words per min	Entities Per Sentence	Entity Density
User 1	Random	5.53	12.24	67.70	1.44	0.24
	CAUSE	1.97	27.00	53.30	3.99	0.34
User 2	Random	5.55	11.25	62.44	1.35	0.27
	CAUSE	2.01	26.98	54.33	3.95	0.37

## Discussion

This is the first study that integrates AL with annotation processes to build clinical NER systems and evaluates it in a real-world task by engaging users. Although many previous AL studies showed substantial savings of annotation in terms of number of samples in simulation, our real world experiments showed that current AL methods did not guarantee savings of annotation time for all users in practice.

This finding could be due to multiple reasons. First, although AL selected more informative sentences and required fewer sentences for building NER models, it often selects longer sentences with more entities, which take a longer time to annotate. According to Table 6 and Table 7, users annotated ~240 sentences queried by *CAUSE* in 120 min (~2.0 sentences per minute) versus ~660 sentences by *Random* in the same time (~5.5 sentences per minute). Our results suggest that the increased information content of actively selected sentences is strongly offset by the increased time required to annotate them. Moreover, it seems that users may have different behaviors for sentences selected by different methods. For example, it seemed that users read randomly sampled sentences faster (62–68 words per minute) than AL selected sentences (53–54 words per minute). All these results demonstrate that AL in practice could be very different from simulation studies and it is critical to benchmark AL algorithms using real-world practical measurements (such as annotation time), instead of theoretical measurements (such as the number of training sentences and the number of words in training sentences).

Active LEARNER system provides gap-free annotation experience so that annotation and model training are running in parallel. However, the active learning performance may be discounted when the model training process is long, especially for CAUSE model when longer sentences are queried. One way to improve the system is that, instead of using the current CRF package that needs to retrain entire dataset each time, we could adapt online learning CRF labeling module with incremental model update to save training time while increasing the update frequency during user annotation. In addition, the performance of the CAUSE model also relied on the quality of clustering results. We did not apply a systematic parameter tuning strategy to find an optimal parameter setting (e.g. optimal number of semantic topics and clusters). The clustering results were not quantitatively evaluated and optimized in our study.

Although the results in this user study showed that the current AL methods could not be guaranteed to save annotation time, compared to passive learning, we gained valuable information about why it happened. If the querying algorithm accounts for the actual annotation time in the model, we believe AL could perform better. Therefore, the next phase of our work will include improving our AL algorithms against the practical measures (i.e., annotation time). One of our plans is to use annotation data collected in this study to develop regression models, which can more accurately estimate annotation time of unlabeled sentences, thus optimizing the AL algorithms for actual annotation time instead of number of samples.

## Conclusions

In this study, we developed the first AL-enabled annotation system for building clinical NER models, which supports the user study to evaluate the actual performance of AL in practice. The user study results indicate that the best effective AL algorithm in the simulation study could not be guaranteed to save actual annotation cost in practice. This could be due to that we did not consider the cost in the model. In the future, we will continue to develop better AL algorithms with the accurate estimation of annotation time and conduct larger scale of user study.
